# Redefining Critical Thinking: Teaching Students to Think like Scientists

**DOI:** 10.3389/fpsyg.2017.00459

**Published:** 2017-03-29

**Authors:** Rodney M. Schmaltz, Erik Jansen, Nicole Wenckowski

**Affiliations:** Department of Psychology, MacEwan UniversityEdmonton, AB, Canada

**Keywords:** scientific thinking, critical thinking, teaching resources, skepticism, education policy

From primary to post-secondary school, critical thinking (CT) is an oft cited focus or key competency (e.g., DeAngelo et al., 2009; California Department of Education, 2014; Alberta Education, 2015; Australian Curriculum Assessment and Reporting Authority, n.d.). Unfortunately, the definition of CT has become so broad that it can encompass nearly anything and everything (e.g., Hatcher, 2000; Johnson and Hamby, 2015). From discussion of Foucault, critique and the self (Foucault, 1984) to Lawson's (1999) definition of CT as the ability to evaluate claims using psychological science, the term critical thinking has come to refer to an ever-widening range of skills and abilities. We propose that educators need to clearly define CT, and that in addition to teaching CT, a strong focus should be placed on teaching students how to think like scientists. Scientific thinking is the ability to generate, test, and evaluate claims, data, and theories (e.g., Bullock et al., 2009; Koerber et al., 2015). Simply stated, the basic tenets of scientific thinking provide students with the tools to distinguish good information from bad. Students have access to nearly limitless information, and the skills to understand what is misinformation or a questionable scientific claim is crucially important (Smith, 2011), and these skills may not necessarily be included in the general teaching of critical thinking (Wright, 2001).

This is an issue of more than semantics. While some definitions of CT include key elements of the scientific method (e.g., Lawson, 1999; Lawson et al., 2015), this emphasis is not consistent across all interpretations of CT (Huber and Kuncel, 2016). In an attempt to provide a comprehensive, detailed definition of CT, the American Philosophical Association (APA), outlined six CT skills, 16 subskills, and 19 dispositions (Facione, 1990). Skills include interpretation, analysis, and inference; dispositions include inquisitiveness and open-mindedness.^1^From our perspective, definitions of CT such as those provided by the APA or operationally defined by researchers in the context of a scholarly article (e.g., Forawi, 2016) are not problematic—the authors clearly define what they are referring to as CT. Potential problems arise when educators are using different definitions of CT, or when the banner of CT is applied to nearly any topic or pedagogical activity. Definitions such as those provided by the APA provide a comprehensive framework for understanding the multi-faceted nature of CT, however the definition is complex and may be difficult to work with at a policy level for educators, especially those who work primarily with younger students.

The need to develop scientific thinking skills is evident in studies showing that 55% of undergraduate students believe that a full moon causes people to behave oddly, and an estimated 67% of students believe creatures such as Bigfoot and Chupacabra exist, despite the lack of scientific evidence supporting these claims (Lobato et al., 2014). Additionally, despite overwhelming evidence supporting the existence of anthropogenic climate change, and the dire need to mitigate its effects, many people still remain skeptical of climate change and its impact (Feygina et al., 2010; Lewandowsky et al., 2013). One of the goals of education is to help students foster the skills necessary to be informed consumers of information (DeAngelo et al., 2009), and providing students with the tools to think scientifically is a crucial component of reaching this goal. By focusing on scientific thinking in conjunction with CT, educators may be better able design specific policies that aim to facilitate the necessary skills students should have when they enter post-secondary training or the workforce. In other words, students should leave secondary school with the ability to rule out rival hypotheses, understand that correlation does not equal causation, the importance of falsifiability and replicability, the ability to recognize extraordinary claims, and use the principle of parsimony (e.g., Lett, 1990; Bartz, 2002).

Teaching scientific thinking is challenging, as people are vulnerable to trusting their intuitions and subjective observations and tend to prioritize them over objective scientific findings (e.g., Lilienfeld et al., 2012). Students and the public at large are prone to naïve realism, or the tendency to believe that our experiences and observations constitute objective reality (Ross and Ward, 1996), when in fact our experiences and observations are subjective and prone to error (e.g., Kahneman, 2011). Educators at the post-secondary level tend to prioritize scientific thinking (Lilienfeld, 2010), however many students do not continue on to a post-secondary program after they have completed high school. Further, students who are told they are learning critical thinking may believe they possess the skills to accurately assess the world around them. However, if they are not taught the specific skills needed to be scientifically literate, they may still fall prey to logical fallacies and biases. People tend to underestimate or not understand fallacies that can prevent them from making sound decisions (Lilienfeld et al., 2001; Pronin et al., 2004; Lilienfeld, 2010). Thus, it is reasonable to think that a person who has not been adequately trained in scientific thinking would nonetheless consider themselves a strong critical thinker, and therefore would be even less likely consider his or her own personal biases. Another concern is that when teaching scientific thinking there is always the risk that students become overly critical or cynical (e.g., Mercier et al., 2017). By this, a student may be skeptical of nearly all findings, regardless of the supporting evidence. By incorporating and focusing on cognitive biases, instructors can help students understand their own biases, and demonstrate how the rigor of the scientific method can, at least partially, control for these biases.

Teaching CT remains controversial and confusing for many instructors (Bensley and Murtagh, 2012). This is partly due to the lack of clarity in the definition of CT and the wide range of methods proposed to best teach CT (Abrami et al., 2008; Bensley and Murtagh, 2012). For instance, Bensley and Spero (2014) found evidence for the effectiveness of direct approaches to teaching CT, a claim echoed in earlier research (Abrami et al., 2008; Marin and Halpern, 2011). Despite their positive findings, some studies have failed to find support for measures of CT (Burke et al., 2014) and others have found variable, yet positive, support for instructional methods (Dochy et al., 2003). Unfortunately, there is a lack of research demonstrating the best pedagogical approaches to teaching scientific thinking at different grade levels. More research is needed to provide an empirically grounded approach to teach scientific thinking, and there is also a need to develop evidence based measures of scientific thinking that are grade and age appropriate. One approach to teaching scientific thinking may be to frame the topic in its simplest terms—the ability to “detect baloney” (Sagan, 1995).

Sagan (1995) has promoted the tools necessary to recognize poor arguments, fallacies to avoid, and how to approach claims using the scientific method. The basic tenets of Sagan's argument apply to most claims, and have the potential to be an effective teaching tool across a range of abilities and ages. Sagan discusses the idea of a baloney detection kit, which contains the “tools” for skeptical thinking. The development of “baloney detection kits” which include age-appropriate scientific thinking skills may be an effective approach to teaching scientific thinking. These kits could include the style of exercises that are typically found under the banner of CT training (e.g., group discussions, evaluations of arguments) with a focus on teaching scientific thinking. An empirically validated kit does not yet exist, though there is much to draw from in the literature on pedagogical approaches to correcting cognitive biases, combatting pseudoscience, and teaching methodology (e.g., Smith, 2011). Further research is needed in this area to ensure that the correct, and age-appropriate, tools are part of any baloney detection kit.

Teaching Sagan's idea of baloney detection in conjunction with CT provides educators with a clear focus—to employ a pedagogical approach that helps students create sound and cogent arguments while avoiding falling prey to “baloney”. This is not to say that all of the information taught under the current banner of “critical thinking” is without value. In fact, many of the topics taught under the current approach of CT are important, even though they would not fit within the framework of some definitions of critical thinking. If educators want to ensure that students have the ability to be accurate consumers of information, a focus should be placed on including scientific thinking as a component of the science curriculum, as well as part of the broader teaching of CT.

Educators need to be provided with evidence-based approaches to teach the principles of scientific thinking. These principles should be taught in conjunction with evidence-based methods that mitigate the potential for fallacious reasoning and false beliefs. At a minimum, when students first learn about science, there should also be an introduction to the basics tenets of scientific thinking. Courses dedicated to promoting scientific thinking may also be effective. A course focused on cognitive biases, logical fallacies, and the hallmarks of scientific thinking adapted for each grade level may provide students with the foundation of solid scientific thinking skills to produce and evaluate arguments, and allow expansion of scientific thinking into other scholastic areas and classes. Evaluations of the efficacy of these courses would be essential, along with research to determine the best approach to incorporate scientific thinking into the curriculum.

If instructors know that students have at least some familiarity with the fundamental tenets of scientific thinking, the ability to expand and build upon these ideas in a variety of subject specific areas would further foster and promote these skills. For example, when discussing climate change, an instructor could add a brief discussion of why some people reject the science of climate change by relating this back to the information students will be familiar with from their scientific thinking courses. In terms of an issue like climate change, many students may have heard in political debates or popular culture that global warming trends are not real, or a “hoax” (Lewandowsky et al., 2013). In this case, only teaching the data and facts may not be sufficient to change a student's mind about the reality of climate change (Lewandowsky et al., 2012). Instructors would have more success by presenting students with the data on global warming trends as well as information on the biases that could lead some people reject the data (Kowalski and Taylor, 2009; Lewandowsky et al., 2012). This type of instruction helps educators create informed citizens who are better able to guide future decision making and ensure that students enter the job market with the skills needed to be valuable members of the workforce and society as a whole.

By promoting scientific thinking, educators can ensure that students are at least exposed to the basic tenets of what makes a good argument, how to create their own arguments, recognize their own biases and those of others, and how to think like a scientist. There is still work to be done, as there is a need to put in place educational programs built on empirical evidence, as well as research investigating specific techniques to promote scientific thinking for children in earlier grade levels and develop measures to test if students have acquired the necessary scientific thinking skills. By using an evidence based approach to implement strategies to promote scientific thinking, and encouraging researchers to further explore the ideal methods for doing so, educators can better serve their students. When students are provided with the core ideas of how to detect baloney, and provided with examples of how baloney detection relates to the real world (e.g., Schmaltz and Lilienfeld, 2014), we are confident that they will be better able to navigate through the oceans of information available and choose the right path when deciding if information is valid.

## Author contribution

RS was the lead author and this paper, and both EJ and NW contributed equally.

### Conflict of interest statement

The authors declare that the research was conducted in the absence of any commercial or financial relationships that could be construed as a potential conflict of interest.

## Footnotes

^1^There is some debate about the role of dispositional factors in the ability for a person to engage in critical thinking, specifically that dispositional factors may mitigate any attempt to learn CT. The general consensus is that while dispositional traits may play a role in the ability to think critically, the general skills to be a critical thinker can be taught (Niu et al., 2013; Abrami et al., 2015).
